# Radiation-Induced Coronary Artery Disease and Its Treatment: A Quick Review of Current Evidence

**DOI:** 10.1155/2018/8367268

**Published:** 2018-10-16

**Authors:** Christopher DeZorzi

**Affiliations:** University of Iowa Hospitals and Clinics, 200 Hawkins Drive, Iowa City, IA 52242, USA

## Abstract

As advances in medical technology arise and the availability of cancer treatment increases, an increased number of patients are receiving cancer treatment. Radiation therapy has evolved to become one of the cornerstones of treatment for various types of cancers. One of the long-term consequences of radiation therapy is radiation-induced coronary artery disease (RICAD). Although the pathophysiology of RICAD may be slightly different and more acute onset than the commonly seen “generic” coronary artery disease, it is common practice to treat RICAD in the same method as nonradiation-induced CAD. This paper summarizes the current research available on the topic and shows there is not enough research to obtain significant data about outcomes and restenosis rates of PCI or outcomes of CABG in RICAD. The aim of this review is to create a concise and easy-to-follow review of the relevant data regarding RICAD and hopefully spark further interest in future studies in this field.

## 1. Introduction

Cardiovascular disease and cancer continue to constitute a major portion of healthcare in the United States. As we continue to develop advances in medical technologies, more patients are receiving radiation therapy and more patients are surviving radiation therapy to suffer from the adverse cardiovascular effects of radiation later in life. With the help of the University of Iowa Library, a thorough search was completed, and evidence was collected using MEDLINE and PubMed using the search terms “coronary artery disease” and “radiation.” This paper summarizes the current research available on the topic with an aim to review the disease condition and create a concise and easy-to-follow review of the relevant data regarding outcomes of PCI and CABG in RICAD.

## 2. Background

**Figure 1:**
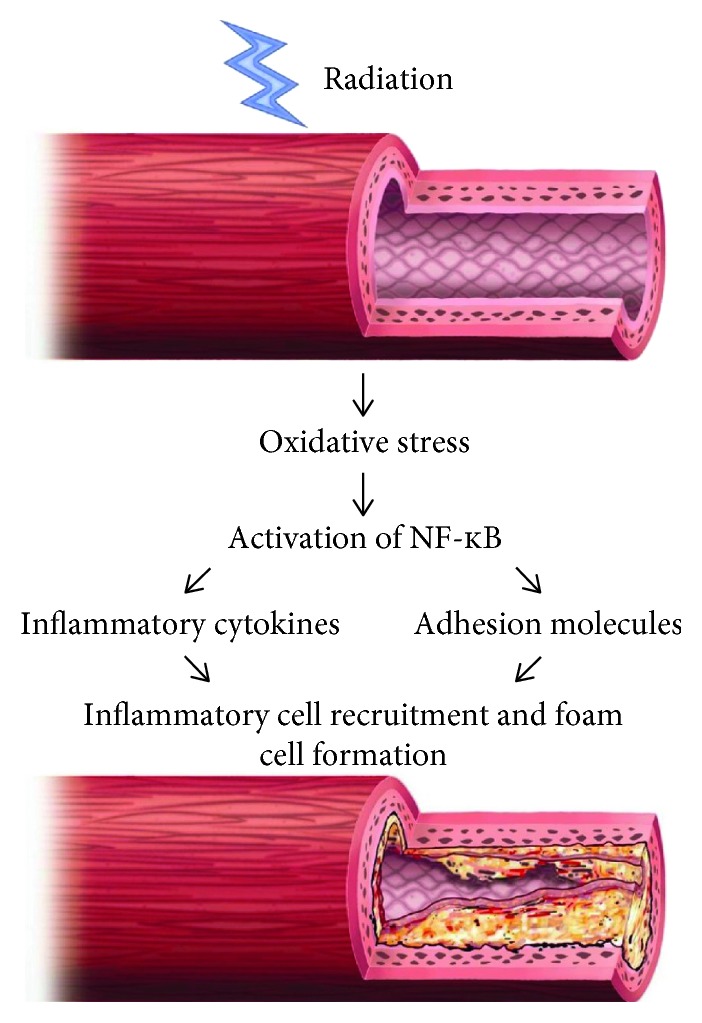
Proposed mechanism of involvement of NF-κB in radiation-induced vascular disease (reproduced from [21]).

The pathology related to radiation-induced coronary artery disease has been described in several articles. The consensus is that, after radiation therapy has occurred, cytokines and adhesion molecules recruit inflammatory cells into the area (Figure 1). The NF-κB pathway has long been considered a prototypical proinflammatory signaling pathway, largely based on the role of NF-κB in the expression of proinflammatory genes including cytokines, chemokines, and adhesion molecules. Patients who have had radiation therapy have chronic inflammation by upregulation of this NF-κB pathway, causing long-term effects of oxidative stress [1]. The inflammatory cell infiltration into the area can be seen after doses as low as 5 Gy, and the proinflammatory state in those receiving radiation therapy potentiates coronary artery disease to develop. Myocardial infarction is the most common cause of cardiac mortality due to radiation [2, 3].

## 3. Material Content

### 3.1. Presentation

The exact incidence of radiation-induced coronary artery disease is difficult to ascertain given the high prevalence of CAD in the general population. Women treated with adjuvant radiation after mastectomy for left-sided breast cancer have been shown in numerous studies to have an increased incidence of fatal coronary vascular disease [4–9]. A recent meta-analysis showed that, for patients with and without radiotherapy, the RRs were 1.30 for coronary heart disease and 1.38 for cardiac mortality [10]. Compared to women receiving treatment for right-sided breast cancer, the relative risk of fatal MI after radiation therapy close to the heart is as high as 2.2 [8]. Further evaluation has revealed that the increased risk appears to be especially prevalent in those who received the highest dose of cardiac radiation and women with left-sided malignancy whose radiation fields included the internal mammary glands [6]. Radiotherapy for breast cancer is associated with an absolute risk increase of 76.4 cases of coronary heart disease and 125.5 cases of cardiac death per 100,000 person‐years [10].

The clinical presentation of radiation-induced coronary artery disease is similar to that of coronary artery disease in the general population but not identical. The more common and usual clinical presentation of coronary artery disease consists of increasing chest pain and shortness of breath associated with exertion. This chest pain, angina pectoris, is a retrosternal chest pressure that typically radiates down the left arm. Angina pectoris is exacerbated with exercise but quickly resolves with rest or nitrate therapy. When the angina is new, brought on by less activity, more severe, more frequent, or occurs at rest, then it is termed “unstable angina” and is suggestive of unstable plaque.

There are several differences that occur in those who have a history of radiation therapy. First, the clinical presentation may be different, as patients with RICAD more commonly have asymptomatic or “silent” myocardial infarctions [11] which can be attributed to the increased amount of damaged nerve endings from their radiation therapy [12]. The younger age at which patients present with CAD makes the clinical presentation very different in RICAD patients as well. The mean age of presentation in these patients is 48 years [13]. Children as young as 12 years of age have suffered from fatal myocardial infarctions [14], and it seems that an increased risk of fatal coronary artery disease may occur as early as 5 years after therapy [6]. Other studies have reported the mean time from chest irradiation until time of presentation is 16 years [13]. Lastly, the clinical presentation of RICAD can be different in the location of the lesions within the coronary arteries. If a patient with a history of radiation gets a coronary angiogram, there are a few differences that can help determine if the lesion was secondary to age-related atherosclerosis or was radiation-induced. Patients with a history of radiation are more likely to have right ostial lesions [15, 16] and a higher incidence of left main disease [13, 17]. Coronary angiogram may also reveal diffuse fibrointimal hyperplasia of coronary vessels [18]. All of these have been implicated as a cause of sudden cardiac death in these patients.

The presentation of RICAD on a subclinical level is also different than the common presentation of coronary artery disease. The basement membrane of capillary walls is thickened as a result of collagen deposition and fibrosis, eventually leading to small vessel occlusion. These lesions resemble atheromatous plaques but differ in their location as described previously and can occur more spontaneously in small arteries [19]. RICAD patients have more severe proximal lesions with fibrous tissue replacing the smooth muscle in the media and adventitia [1, 14, 17, 20, 21].

### 3.2. Prevention

The best model for protecting against RICAD is avoiding erroneous radiation to the heart altogether. Chest radiation treatments will remain a mainstay in cancer treatment, so modern radiation therapy avoiding the heart is crucial for obtaining better long-term outcomes. Modern radiotherapy is associated with lower cardiac morbidity due to limited penetration by the electron beam, careful planning to avoid the heart, and cardiac shielding [19, 22, 23]. These advancements in the knowledge of preventing RICAD have led to clinical improvements. Breast cancer patients diagnosed before 1980 compared with breast cancer patients diagnosed after 1980 have a RR of coronary heart disease of 1.32 [10]. Similar to most diseases, exposure to an increased number of risk factors correlates with a higher likelihood of RICAD. Risk factors for RICAD include a higher dose of radiation (>30–35 Gy), higher dose/fraction (>2 Gy/day), increased field size (especially increased exposure of the heart), proximity of the tumor to the heart, younger age at exposure, cardiotoxic chemotherapy regiments, and classic CAD risk factors including tobacco use and diabetes.

**Figure 2:**
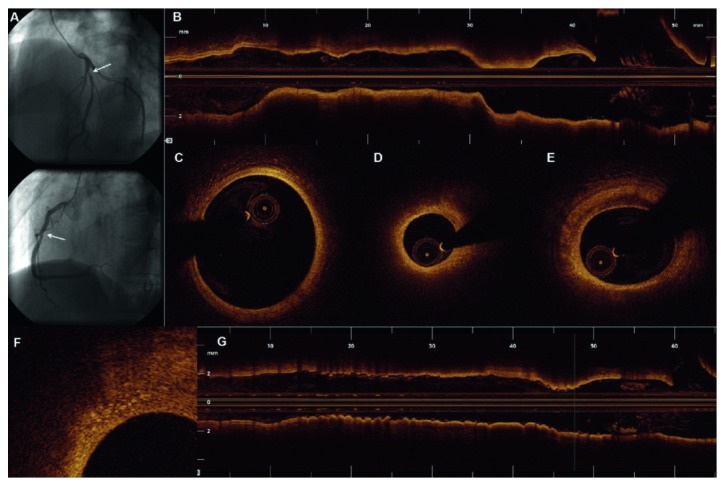
Coronary angiography and subsequent OCT evaluation of LAD (reproduced from [24]).

Although we are using safer techniques, more people are receiving treatment for cancer, and numerous case reports of RICAD are present. An example to help illustrate RICAD is a previously published case study of a 31-year-old woman studied with optical coherence tomography who presented to the ED with chest pain and sudden onset of dyspnea secondary to myocardial infarction. She had been diagnosed with nodular sclerosing Hodgkin's lymphoma three years prior and had received six cycles of Adriamycin, bleomycin, vinblastine, and dacarbazine. Coronary angiography revealed mild stenosis of the mid-right coronary artery and severe focal stenosis of the proximal left anterior descending coronary artery (LAD) which was further evaluated by OCT for complete anatomical characterization. Segments proximal and distal to the lesion had normal wall characteristics, with a typical 3-layer structure in almost all the vessel circumference. The lesion itself presented a minimal diameter of 1.6 mm and a minimal luminal area of 2.04 mm^2^. OCT revealed that the plaque had heterogeneous morphologic features with fibrous characteristics (hyperintense and relatively homogeneous) and areas with changes suggesting the presence of lipid deposits (Figure 2). Macrophages were also seen in the boundary between a fibrous cap and the top of a necrotic core. The fact that these lesions, despite fibrous characteristics, had some lipid components represents an overlap of RICAD with age-related atherosclerosis [24].

### 3.3. Treatment

It is common practice to treat RICAD lesions similar to those found in the general population. Percutaneous coronary intervention (PCI) with bare metal or drug-eluding stents, coronary artery bypass surgery, and pharmacologic-only management are all options used. Pharmacologic-only treatment is much less common, and there are no data to suggest how this approach is compared to PCI and CABG. The decision between PCI and CABG depends on several factors including anatomy of the lesion(s) present and the surgical risk associated with the patient. CABG is a seemingly good option given these patients are usually younger and have less comorbidities, but the mediastinal fibrosis present in the majority of patients who receive anterior irradiation can make surgery more difficult than in patients without prior radiation therapy. In one study [25], the internal mammary artery was an amenable option and routinely harvested less than one-half the time. Radiation-associated heart disease has increased long-term mortality after cardiac surgery, and some investigators have suggested that alternative treatment strategies may be more appropriate [26].

Percutaneous coronary revascularization technology is much less invasive especially for isolated, nonostial coronary artery lesions. PCI offers a valuable option in the management of even complex lesions as the first-line intervention or as an alternative when bypass surgery is difficult [11, 27–29]. Although this is the standard of care, the restenosis rate of PCI in RICAD patients compared to the general population is unknown. Early studies indicated that percutaneous angioplasty had a higher rate of restenosis [29]. One study [30] followed up 15 lymphoma patients, treated with thoracic radiation, for 10 years after receiving bare metal coronary stents secondary to symptomatic coronary artery disease. They found that, in their radiation-treated cohort, patients had an 86% incidence of coronary restenosis after coronary stenting. Another study [31] used more contemporary practice methods, including placing drug-eluting stents, and showed similar restenosis rates after PCI between a control group and patients with a history of external beam radiation therapy. Both studies were limited by their total number of patients, and follow-up studies are needed to support findings in either direction. The overall mortality of RICAD patients who receive PCI is also unclear. Although the previously discussed study [31] only followed up the patients for 3 years after PCI, there were no significant differences in myocardial infarction, cardiac death, or overall mortality. In contrast, another study [32] did show long-term PCI outcomes of higher mortality in patients with RICAD.

There is no direct comparison between PCI and CABG or data to suggest how other less common treatment options including pharmacologic-only treatment are compared with PCI or CABG.

## 4. Conclusion

The presentation of radiation-induced coronary artery disease includes younger patients with occasionally silent disease who present with proximal, ostial, left main, and sometimes diffuse lesions. The underlying pathology is slightly different from age-related atherosclerosis and involves the upregulation of the NF-κB proinflammatory pathway. The current treatment of RICAD is comparable with other forms of CAD, but the outcomes of these treatment measures have not been fully studied. Outcomes and restenosis may be similar to those of the general population, or the difference in pathophysiology could be enough to make the restenosis rate increased. As modern radiation therapies improve prevention of RICAD, less CAD in patients who have a history of radiation therapy will be attributable to their radiation, and therefore, the treatment and restenosis rates will be similar.

To date, there are no unifying studies to suggest the most appropriate evidence-based treatment for this subpopulation of patients. Patients with RICAD will need individualized treatment from physicians experienced in RICAD. Ideally, patients can be consulted on the risks and benefits of treatment with CABG vs PCI, and informed decision-making can be made with the limited information that is available. The developing field of cardio-oncology helps patients receive multidisciplinary care from high-quality centers and should help cancer patients receive higher quality cardiovascular care.

## References

[1] Brosius F. C., Waller B. F., Roberts W. C. (1981). Radiation heart disease. Analysis of 16 young (aged 15 to 33 years) necropsy patients who received over 3,500 rads to the heart. *American Journal of Medicine*.

[2] Glanzmann C., Kaufmann P., Jenni R., Hess O. M., Huguenin P. (1998). Cardiac risk after mediastinal irradiation for Hodgkin’s disease. *Radiotherapy and Oncology*.

[3] Hoppe R. T. (1997). Hodgkin’s disease: complications of therapy and excess mortality. *Annals of Oncology*.

[4] Cuzick J., Stewart H., Rutqvist L. (1994). Cause-specific mortality in long-term survivors of breast cancer who participated in trials of radiotherapy. *Journal of Clinical Oncology*.

[5] Early Breast Cancer Trialists’ Collaborative Group (2000). Favourable and unfavourable effects on long-term survival of radiotherapy for early breast cancer: an overview of the randomised trials. *Lancet*.

[6] Gyenes G., Erik Rutqvist L., Liedberg A., Fornander T. (1998). Long-term cardiac morbidity and mortality in a randomized trial of pre- and postoperative radiation therapy versus surgery alone in primary breast cancer. *Radiotherapy and Oncology*.

[7] Jones J. M., Ribeiro G. G. (1989). Mortality patterns over 34 years of breast cancer patients in a clinical trial of post-operative radiotherapy. *Clinical Radiology*.

[8] Paszat L. F., Mackillop W. J., Groome P. A., Boyd C., Schulze K., Holowaty E. (1998). Mortality from myocardial infarction after adjuvant radiotherapy for breast cancer in the surveillance, epidemiology, and end-results cancer registries. *Journal of Clinical Oncology*.

[9] Rutqvist L. E., Johansson H. (1990). Mortality by laterality of the primary tumour among 55,000 breast cancer patients from the Swedish Cancer Registry. *British Journal of Cancer*.

[10] Cheng Y. J., Nie X.-Y., Ji C.-C. (2017). Long-term cardiovascular risk after radiotherapy in women with breast cancer. *Journal of the American Heart Association*.

[11] Om A., Vetrovec G. W. (1993). Coronary angioplasty of radiation-induced stenosis. *American Heart Journal*.

[12] Adams M. J., Hardenbergh P. H., Constine L. S., Lipshultz S. E. (2003). Radiation-associated cardiovascular disease. *Critical Reviews in Oncology/Hematology*.

[13] McEniery P. T., Dorosti K., Schiavone W. A., Pedrick T. J., Sheldon W. C. (1987). Clinical and angiographic features of coronary artery disease after chest irradiation. *American Journal of Cardiology*.

[14] Uriel N., Vainrib A., Jorde U. P. (2010). Mediastinal radiation and adverse outcomes after heart transplantation. *Journal of Heart and Lung Transplantation*.

[15] Nakhjavan F. K., Yazdanfar S., Friedman A. (1984). Percutaneous transluminal coronary angioplasty for stenosis of the ostium of the right coronary artery after irradiation for Hodgkin’s disease. *American Journal of Cardiology*.

[16] Orzan F., Brusca A., Conte M. R., Presbitero P., Figliomeni M. C. (1993). Severe coronary artery disease after radiation therapy of the chest and mediastinum: clinical presentation and treatment. *Heart*.

[17] Levine G. N., Bates E. R., Blankenship J. C. (2011). ACCF/AHA/SCAI Guideline for percutaneous coronary intervention: executive summary: a report of the American College of Cardiology Foundation/American Heart Association Task Force on practice guidelines and the society for cardiovascular angiography and interventions. *Circulation*.

[18] Yusuf S. W., Sami S., Daher I. N. (2011). Radiation-induced heart disease: a clinical update. *Cardiology Research and Practice*.

[19] Gaya A. M., Ashford R. F. (2005). Cardiac complications of radiation therapy. *Clinical Oncology*.

[20] Verin V., Popowski Y., de Bruyne B. (2001). Endoluminal beta-radiation therapy for the prevention of coronary restenosis after balloon angioplasty. The Dose-Finding Study Group. *New England Journal of Medicine*.

[21] Weintraub N. L., Jones W. K., Manka D. (2010). Understanding radiation-induced vascular disease. *Journal of the American College of Cardiology*.

[22] Nixon A. J., Manola J., Gelman R. (1998). No long-term increase in cardiac-related mortality after breast-conserving surgery and radiation therapy using modern techniques. *Journal of Clinical Oncology*.

[23] Vallis K. A., Pintilie M., Chong N. (2002). Assessment of coronary heart disease morbidity and mortality after radiation therapy for early breast cancer. *Journal of Clinical Oncology*.

[24] Caro-Codon J., Jiménez-Valero S., Galeote G., Sanchez-Recalde A., Moreno R. (2016). Radiation-induced coronary artery disease: useful insights from OCT. *International Journal of Cardiology*.

[25] Chang A. S., Smedira N. G., Chang C. L. (2007). Cardiac surgery after mediastinal radiation: extent of exposure influences outcome. *Journal of Thoracic and Cardiovascular Surgery*.

[26] Annest L. S., Anderson R. P., Li W., Hafermann M. D. (1983). Coronary artery disease following mediastinal radiation therapy. *Journal of Thoracic and Cardiovascular Surgery*.

[27] Glanzmann C., Huguenin P., Lütolf U. M., Maire R., Jenni R., Gumppenberg V. (1994). Cardiac lesions after mediastinal irradiation for Hodgkin’s disease. *Radiotherapy and Oncology*.

[28] Lee P. J., Mallik R. (2005). Cardiovascular effects of radiation therapy: practical approach to radiation therapy-induced heart disease. *Cardiology in Review*.

[29] Veeragandham R. S., Goldin M. D. (1998). Surgical management of radiation-induced heart disease. *Annals of Thoracic Surgery*.

[30] Schomig K., Ndrepepa G., Mehilli J., Pache J., Kastrati A., Schömig A. (2007). Thoracic radiotherapy in patients with lymphoma and restenosis after coronary stent placement. *Catheterization and Cardiovascular Interventions*.

[31] Liang J. J., Sio T. T., Slusser J. P. (2014). Outcomes after percutaneous coronary intervention with stents in patients treated with thoracic external beam radiation for cancer. *JACC: Cardiovascular Interventions*.

[32] Reed G. W., Masri A., Griffin B. P., Kapadia S. R., Ellis S. G., Desai M. Y. (2016). Long-term mortality in patients with radiation-associated coronary artery disease treated with percutaneous coronary intervention. *Circulation: Cardiovascular Interventions*.

